# Heat-load-dependent inbreeding depression for production traits in Italian Holstein cattle

**DOI:** 10.1186/s12711-026-01069-2

**Published:** 2026-07-19

**Authors:** Francesco Tiezzi, Joao Claudio do Carmo Panetto, Simone Callegaro, Jicai Jiang, Andrea Zanotti, Michela Ablondi, Claudio Cipolat-Gotet, Raffaella Finocchiaro, Martino Cassandro, Johannes Van Kaam, Christian Maltecca

**Affiliations:** 1 Department of Agriculture, Food, Environment and Forestry, University of Florence, 50144 Florence, Italy; 2 Embrapa Dairy Cattle, Juiz de Fora, 36038-330 Brazil; 3 Animal Science Department, North Carolina State University, Raleigh, NC 27695 USA; 4 Department of Veterinary Science, University of Parma, 43126 Parma, Italy; 5 Italian Holstein, Brown Swiss and Jersey National Breeders Association (ANAFIBJ), 26100 Cremona, Italy; 6 Department of Agronomy, Food, Natural Resources, Animals and Environment, University of Padua, 35020 Legnaro, Italy

## Abstract

**Background:**

The objective of this study was to test whether the negative effects of inbreeding on production traits varied according to the level of environmental load. Traits analyzed were milk, fat, and protein yields, and somatic cell score (SCS). Environmental conditions were described using temperature (TEMP), relative humidity (RH), and the temperature–humidity index (THI), each divided into five equally sized classes. For each trait, the environmental variable with the largest impact was used to evaluate inbreeding effects across its classes. Inbreeding was measured using pedigree (F_PED_), the diagonal of the genomic relationship matrix (F_GRM_), and runs of homozygosity (F_ROH_).

**Results:**

Genomic-based inbreeding measures resulted in larger estimated inbreeding effects, compared to pedigree measures, with F_GRM_ and F_ROH_ showing similar results. RH most affected milk yield, with losses of ~ 600 g/day in the highest RH class. Inbreeding led to losses of ~ 100 g/day per 1% increase, with genomic measures scaled to match F_PED_. For milk yield, losses were ~ 20% greater under higher environmental stress. THI was the most impactful variable for fat yield, with losses of ~ 90 g/day in the highest THI category. Inbreeding-related losses increased in more stressful environments, with average reductions of 3 g/day per 1% inbreeding, reaching 4 g/day in the highest THI class. Protein yield and SCS were mainly influenced by TEMP, with losses of ~ 70 g/day of protein and increases of 0.06 SCS units. Inbreeding caused losses of ~ 3.5 g/day in protein yield and increases of ~ 0.006 SCS units per 1% inbreeding. For these two traits, the relationship between inbreeding effects and environmental stress was less clear. For milk and fat yields, there was a significant interaction between environmental and inbreeding effects, suggesting that inbreeding depression influences heat tolerance in dairy cows.

**Conclusions:**

For milk and fat yields, there was a significant interaction between environmental and inbreeding effects, indicating that environmental conditions modulate the expression of inbreeding depression. These findings suggest that inbreeding depression influences heat tolerance in dairy cows.

**Supplementary Information:**

The online version contains supplementary material available at 10.1186/s12711-026-01069-2.

## Background

Climatic conditions vary seasonally, and recent decades have seen a documented increase in global temperatures [1, 2]. These environmental changes pose significant challenges to dairy herds, which must sustain reproduction and production while coping with increasingly stressful conditions, often characterized as an environmental load (EL). Elevated temperatures can substantially affect physiology, metabolism, and overall health of dairy cattle, disrupting energy balance, decreasing feed intake, impairing reproductive function, and ultimately reducing milk production [3, 4].

One promising strategy is animal breeding, with the selection of individuals exhibiting greater resilience to environmental stress emerging as an effective approach to mitigate these effects [5]. However, understanding the biological mechanisms underlying heat resilience remains a critical area for future research. Specifically, clarifying whether observed associations between genetic or physiological traits and resilience are causal, or simply correlative, will be essential for developing targeted breeding and management interventions. Addressing these questions will be key to improving dairy cattle adaptation in the face of ongoing climate change.

Genetic variation for tolerance to heat stress, expressed in the form of genotype-by-environment interactions, has the potential to provide the basis for such selection [6]. These interactions imply that different genotypes respond differently to environmental changes, affecting performance across diverse conditions. Research in cattle and pigs has shown that the genetic component associated with heat stress is present, underlining the potential for selecting animals that are more resilient to environmental challenges [7–9]. For this reason, various initiatives within dairy cattle breeding programs are increasingly focused on addressing this issue. These efforts include conducting studies and genetic evaluations aimed at supporting the selection of germplasm better suited to specific environments, particularly under seasonal heat stress conditions [10–12].

While efforts to breed more heat-tolerant cattle are currently underway, it is important to explore other sources of variation. Dairy cows exhibit differences in their resilience and capacity to realize their full potential under conditions of elevated temperature and humidity that surpass their comfort zone. This variation is partly influenced by additive genetic factors; however, non-additive genetic factors may also play a significant role in shaping these traits.

It is well known that increased inbreeding can reduce production, reproduction, and survival [13], and in Holstein-based dairy herds rising average inbreeding and evidence of inbreeding depression (ID) have been reported across populations and traits [14–16]. Dairy systems are also experiencing more frequent and intense episodes of thermal and humidity stress, yet despite evidence for both phenomena there is a paucity of research dedicated to understanding their interplay. Here we hypothesize that ID may vary with environmental stress rather than act uniformly, and we examined whether inbreeding effects on four key dairy traits, milk yield (MY), fat yield (FY), protein yield (PY), and somatic cell score (SCS), depend on environmental conditions, i.e., whether there is an interaction between inbreeding and environment (F × E). 

Few studies have explored interactions between inbreeding and environment in both natural and captive populations, generally reporting stronger ID under harsh or stressful conditions [17–20]. Experimental work in *Drosophila melanogaster* demonstrated that ID for egg-to-adult viability emerged only at stressful high or low temperatures [21], while field studies in wild bird populations revealed that the negative effects of inbreeding generally increase with environmental stress [22, 23]. Similar patterns have been observed in plants, where drought stress amplified ID in different traits in *Crepis sancta* (*Asteraceae*) [24]. Recent work in red deer (*Cervus elaphus*) showed that inbreeding coefficients and ID vary across space, with individuals born in harsher northern regions exhibiting lower juvenile survival and birth weights compared to southern regions [25], and earlier studies suggest a temperature-dependent inbreeding effects on birth weight [26, 27]. Comparable literature in domesticated species is sparse, but one example was observed in Soay sheep, in which some interactions between inbreeding and environmental variation were reported across six morphological and fitness traits [28], although it was unclear what environmental conditions would exert the largest effects and promote the losses due to inbreeding at the same time. Collectively, these studies indicate that, while ID often intensifies under higher EL, its magnitude and detectability vary across traits, species, and ecological contexts. Inbreeding depression appears to depend on local environmental conditions, highlighting spatially dependent interaction with the environment and making it difficult to benchmark the different ‘environments’ in terms of the ‘stress’ they apply. In other words, it appears difficult to identify the nature and magnitude of EL under which ID would become severely detrimental. Nevertheless, the study of the F × E is mandated by evidence found in wild populations as well as the need to breed more heat tolerant livestock.

While genotype-by-environment interactions have been extensively studied as a source of genetic variation for heat tolerance, the role of F × E remains largely unexplored in livestock. The objective of this study is to assess the interplay between ID and heat stress. We hypothesize that ID may vary with environmental stress rather than act uniformly, reflecting an environment-dependent expression of inbreeding load, which can also be addressed as environmental determination of ID. Here we examined four key dairy traits (milk, fat, and protein yields, and SCS) and how they depend on the F × E conditions. Specifically, the present study aimed: (1) to assess the environmental load on the production traits, expressed as the magnitude of climatic (heat) stress, and (2) to estimate the effects of inbreeding on these traits conditionally on the level of environmental load.

## Methods

### Climate records and production traits

In this study, we evaluated the effects of heat stress and inbreeding on the productive performance of dairy cows. Test-day records for daily MY, FY, PY, and SCS were used, along with pedigree information and inbreeding measures, as provided by the ANAFIBJ (Italian Holstein, Brown, and Jersey Breeders Association) national genetic evaluation system.

The initial dataset included 443,644 records from 24,606 cows recorded on 36,772 herd-test-day groups (HTD). The HTD groups were created by concatenating the herd identification code with the date of herd visit. Data editing procedure included removing test-day records with MY lower than 10 kg and larger than 65 kg, FY greater than 3.5 kg and PY greater than 2.5 kg, negative values of SCS, as well as records with days in milk (DIM) smaller than 5 or larger than 305 days. HTD with less than 3 cows tested were excluded. Mean, standard deviation, minimum and maximum values for each of these four traits are shown in Table 1, while histograms reporting the distribution of each trait analysed are reported in Additional file 2, Figs. S5–8.

**Table 1:** Mean, standard-deviation, minimum and maximum values for each of the four traits

Trait	Mean	SD	Min	Max
Milk yield (kg)	37.90	8.92	10.00	65.00
Fat yield (kg)	1.48	0.42	0.25	3.50
Protein yield (kg)	1.29	0.28	0.26	2.50
Somatic cell score	2.25	1.90	− 3.64	9.97

Mean, SD, minimum (Min), and maximum (Max) values were shown for milk yield (kg), fat yield (kg), protein yield (kg), and somatic cell score (SCS) in the studied Italian Holstein population

Similarly, maximum temperature (TEMP), relative humidity (RH), and Temperature-Humidity Index (THI) values were obtained from the ANAFIBJ genetic evaluation procedure, which was used to generate the heat tolerance index (IHT) [11]. Weather stations were matched to farms based on their location. Using the latitude and longitude coordinates of both stations and farms, the distance between each farm and each weather station was calculated. Candidate stations were required to lie within an 80 km distance and within a 500 m altitude difference. Among candidate stations with measurements on the test-day, those in similar directions are pruned, retaining the closest representative per 60 degree angular sector. The remaining stations are combined into a weighted centroid using inverse-distance weighting, giving greater influence to closer stations. For the dataset used in this study, the average distance to the nearest assigned station was 25 km, while the average distance to the most remote assigned station was 50 km. Once TEMP, RH, and THI values were available for each HTD, quintiles for each variable were calculated. TEMP quintiles were used for creating the TEMPc variable, i.e. five classes of TEMP where records were binned based on the first four quintiles as thresholds. Similarly, RHc was created using RH and THIc was created using THI. This procedure allowed having the five classes with balanced frequencies. This stratification allowed us to assess trait responses and inbreeding effects across a gradient of environmental stress. Finally, only records from cows represented across all TEMPc, RHc, and THIc classes were retained. At the end of the procedure, the dataset included 414,514 records from 24,395 cows recorded on 25,011 HTD groups.

Ten 30 days DIM classes were created, with the last one with an upper bound of 305 DIM. Similarly, lactation numbers beyond the second were grouped, creating three classes (first, second, “third and greater” lactations). Classes of lactation number by lactation stage (LNLS) were created, combining the two fields and generating 30 classes.

### Pedigree, genotypes and inbreeding measures

The complete pedigree file comprised 9,615,703 animals and included information on sire, dam, and year of birth. Of these, 178,049 individuals were retained after pruning. Estimates of individual pedigree inbreeding (F_PED_) [29] were calculated with the method described by [30]. The potential influence of pedigree depth variation on F_PED_ had been previously evaluated in the same population by Panetto et al. [31]. Individual increase in inbreeding coefficients ($\:\varDelta\:{F}_{i}$), as presented by Gutierrez et al. [32], were calculated using the Endog v 4.8 program [33] and tested as an alternative measure to the traditional F_PED_ coefficient ($\:{F}_{i}$) for estimating ID. In that study, the effects obtained using $\:{F}_{i}$ and $\:\varDelta\:{F}_{i}$ were highly consistent in magnitude and direction (results not shown). Because a prior analysis on the same dataset showed that F_i_ and ∆F_i_ yielded highly consistent estimates in both magnitude and direction, only F_i_ was retained in the present study. The mean number of equivalent complete generations (ECG) was calculated using the *optiSel* package in R [34]. The ECG represents the sum of $(1/2)^{n}$ known ancestors for each individual, where * n* is the number of generations separating the individual from a given ancestor. This metric quantifies pedigree depth by accumulating the expected genetic contributions of all known ancestors. Given the relatively deep and homogeneous pedigree structure (8.72 ± 1.34 equivalent complete generations) and the observed absence of meaningful differences between Fi and $\:\varDelta\:{F}_{i}$, we proceeded using only the traditional pedigree-based inbreeding coefficient (hereafter F_PED_).

Quality control procedures were applied to the genomic data to ensure reliability. Animals with a genotype call rate below 95% or a Mendelian inconsistency rate above 1% were excluded, and SNPs with minor allele frequency (MAF) below 0.02 were removed. After quality control, the dataset included 80,810 dairy cows genotyped with 21 different SNP chips. Most animals were genotyped with medium-density chips (≈ 40–70 K markers). Approximately 80% of cows had sires genotyped with high-density chips. All medium-density genotypes were imputed to a common set of 84,445 SNPs preselected by ANAFIBJ, and high-density genotypes were downsampled to the same marker set. Imputation was performed as part of ANAFIBJ’s routine genomic evaluation using an enhanced version of PedImpute [35]; further details are available in [36].

Genomic inbreeding was obtained from the diagonal of the genomic relationship matrix (F_GRM_), following the method 1 proposed by VanRaden [37]. Inbreeding as autozygosity was obtained using runs of homozygosity (F_ROH_), calculated as the proportion between the sum of the ROH segments length per cow and the total length of the autosomal genome covered by SNPs (2.48 Gbp in this study). The ROH segments were detected with the use of the detectRUNS package in R [38] using a sliding window approach, with parameters described by Ablondi et al. [16]: at least 15 SNPs in a run, a minimum length of a run equal to 1 Mb, a maximum distance of 500 kb between consecutive SNPs in a window, a minimum density of 1 SNP per 100 kb, and a maximum of one missing and one heterozygous SNP in a run.

Inbreeding values were retained only for cows with phenotypic information. All inbreeding measures were centered and scaled to have mean equal to 100 and standard deviation equal to 1, for comparison reasons.

### Statistical analyses

#### Selecting environmental indicators

An initial analysis evaluated the effect of environmental indicators on the traits by estimating least squares means for different classes of these indicators and testing their influence on each trait. The model was:

1 $$\:\mathbf{y}={{\mathbf{X}}_{0}\boldsymbol{\upbeta\:}}_{0}+{\mathbf{X}}_{2}{\boldsymbol{\upbeta\:}}_{2}+{\mathbf{Z}}_{\mathbf{h}}\mathbf{h}+{\mathbf{Z}}_{\mathbf{p}}\mathbf{p}+\mathbf{e},$$

where $\:\mathbf{y}$ is a vector containing phenotypic records (MY, FY, PY or SCS); $\:{\mathbf{X}}_{0}$ and $\:{\boldsymbol{\upbeta\:}}_{0}$ are the incidence matrix and vector of effects for the five classes of environmental indicator of choice (TEMPc, RHc or THIc); $\:{\mathbf{X}}_{2}$ and $\:{\boldsymbol{\upbeta\:}}_{2}$ are the incidence matrix and vector of effects for the LNLS effect (30 classes); $\:{\mathbf{Z}}_{\mathbf{h}}$ and $\:\mathbf{h}$ are the incidence matrix and vector of solutions for the HTD random effect, which follows $\:\mathbf{h}\:\sim\:\mathrm{N}\mathrm{I}\mathrm{I}\mathrm{D}(0,{{\upsigma\:}}_{\mathrm{h}}^{2}\mathbf{I})$, where $\:{{\upsigma\:}}_{\mathrm{h}}^{2}$ is the herd test day variance and $\:\mathbf{I}$ is an identity matrix; $\:{\mathbf{Z}}_{\mathbf{p}}$ and $\:\mathbf{p}$ are the incidence matrix and vector of solutions for the cow permanent environmental random effect, which follows $\:\mathbf{p}\:\sim\:\mathrm{N}\mathrm{I}\mathrm{I}\mathrm{D}(0,{{\upsigma\:}}_{\mathrm{p}}^{2}\mathbf{I})$, with variance $\:{{\upsigma\:}}_{\mathrm{p}}^{2}$ ; and $\:\mathbf{e}\:\sim\:\mathrm{N}(0,\:{{\upsigma\:}}_{\mathrm{e}}^{2}\mathbf{I})$ is the vector of residuals, with variance $\:{{\upsigma\:}}_{\mathrm{e}}^{2}$. The model was implemented in R using the “lmer” function of the “lme4” package [39]. The analysis of variance (type 3) table was generated using the native ‘anova’ R function [40]. Least square mean (LSM) estimates were then calculated for the fixed effect of the climate classes using R-package ‘emmeans’ [41], Tukey contrast values and significance were calculated using function ‘cld’ from R-package ‘multcomp’ [42]. Given four traits and three environmental indicators, a total of 12 models were fitted.

#### Variance components and systematic effects estimation

The second step involved implementing single-trait animal models that included the inbreeding effect as a covariate nested within climate classes, along with the additive genetic effect of the animal. For each trait, the models incorporated the effect of either maximum temperature (TEMPc), relative humidity (RHc), or Temperature-Humidity Index (THIc), depending on the results of the analysis of variance.

Specifically, the model had form:

2 $$\:\mathbf{y}={{\mathbf{X}}_{0}\boldsymbol{\upbeta\:}}_{0}+{\mathbf{X}}_{1}{\boldsymbol{\upbeta\:}}_{1}+{\mathbf{X}}_{2}{\boldsymbol{\upbeta\:}}_{2}+{\mathbf{Z}}_{\mathbf{h}}\mathbf{h}+{\mathbf{Z}}_{\mathbf{p}}\mathbf{p}+{\mathbf{Z}}_{\mathbf{a}}\mathbf{a}+\mathbf{e},$$

where $\:\mathbf{y}$ is a vector containing phenotypic records (MY, FY, PY or SCS); $\:{\mathbf{X}}_{1}$ and $\:{\boldsymbol{\upbeta\:}}_{1}$ are the incidence matrix and vector of effects for the inbreeding effect (F_PED_, F_GMR_ or F_ROH_, respectively) nested within the five classes of environmental indicator of choice; $\:{\mathbf{Z}}_{\mathbf{a}}$ and $\:\mathbf{a}$ are the incidence matrix and vector of solutions for the cow additive genetic random effect, which follows $\:\mathbf{a}\:\sim\:\mathrm{N}(0,{{\upsigma\:}}_{\mathrm{a}}^{2}\mathbf{A})$, where $\:{{\upsigma\:}}_{\mathrm{a}}^{2}$ is the genetic variance and $\:\mathbf{A}$ is the pedigree-derived relationship matrix, built ignoring inbreeding to avoid collinearity between additive genetic effects with the inbreeding covariates included in the model. Elements $\:{\mathbf{X}}_{0}$, $\:{\boldsymbol{\upbeta\:}}_{0}$, $\:{\mathbf{X}}_{2}$, $\:{\boldsymbol{\upbeta\:}}_{2}$, $\:{\mathbf{Z}}_{\mathbf{h}}$, $\:\mathbf{h}$, $\:{\mathbf{Z}}_{\mathbf{p}}$, $\:\mathbf{p}$ and $\:\mathbf{e}$ are as defined above. For this model, variance components and fixed effects were estimated under a Bayesian framework using the GIBBSF90+ software, part of the BLUPF90 programs family [43]. Flat priors were used for variance components ($\:{{\upsigma\:}}_{\mathrm{h}}^{2}$, $\:{{\upsigma\:}}_{\mathrm{p}}^{2}$, $\:{{\upsigma\:}}_{\mathrm{a}}^{2}$, $\:{{\upsigma\:}}_{\mathrm{e}}^{2}$), bounded uniform priors were used for the solutions of systematic effects ($\:{\boldsymbol{\upbeta\:}}_{0}$, $\:{\boldsymbol{\upbeta\:}}_{1}$, $\:{\boldsymbol{\upbeta\:}}_{2})$, while normal distribution priors were used for solutions of random effects ($\:\mathbf{h}$, $\:\mathbf{p}$, $\:\mathbf{a}$, $\:\mathbf{e}$). All estimates were obtained using a total of 600,000 Markov Chain Monte Carlo iterations in each analysis with a burn-in of 100,000 and a thinning period of 50 iterations. The posterior mean of each parameter, computed after burn-in, was taken as the point estimate, and the posterior standard deviation as the associated measure of uncertainty. Convergence was assessed by visual inspection of trace plots. The models were first implemented to obtain estimates of variance components ($\:{{\upsigma\:}}_{\mathrm{h}}^{2}$, $\:{{\upsigma\:}}_{\mathrm{p}}^{2}$, $\:{{\upsigma\:}}_{\mathrm{a}}^{2}$, $\:{{\upsigma\:}}_{\mathrm{e}}^{2}$), once these were obtained, the Gibbs sampler was run again with fixed values for the variance components, in order to obtain posterior distributions of effects’ vectors ($\:{\boldsymbol{\upbeta\:}}_{0}$, $\:{\boldsymbol{\upbeta\:}}_{1}$, $\:{\boldsymbol{\upbeta\:}}_{2})$, which were then used for subsequent analyses. Additionally, we tested models that (1) admitted heterogeneous residual variance and (2) admitted heterogeneous residual, herd-test-day and additive genetic variance as well as the covariance among environmental classes (i.e. multiple trait models). Since in both cases we didn’t find significant heteroskedasticity of the variances, we proceeded with the simpler model shown above.

The slopes of the inbreeding effects estimated in $\:{\boldsymbol{\upbeta\:}}_{1}$ were also illustrated in plots containing separated lines for each category of environment. In order to obtain prediction of productive performance as a function of inbreeding level under specific environmental conditions, LSM estimates were obtained as:

3 $$\:{\widehat{\mathrm{y}}}_{\mathrm{i}}={{\upbeta\:}}_{0,\mathrm{i}}+{\mathrm{k}}_{1}{{\upbeta\:}}_{1,\mathrm{i}},$$

where $\:{\widehat{\mathrm{y}}}_{\mathrm{i}}$ is the vector of the predicted phenotypic values under the environmental class $\:\mathrm{i}$; $\:{{\upbeta\:}}_{0,\mathrm{i}}$ is the intercept for the environmental class $\:\mathrm{i}$; $\:{\mathbf{k}}_{1}$ is a vector of inbreeding values and $\:{{\upbeta\:}}_{1,\mathrm{i}}$ is the slope for inbreeding effect for class $\:\mathrm{i}$. This procedure takes the incidence matrix and vector of effects for the five classes of environmental indicator of choice together with the incidence matrix and vector of effects for the inbreeding effect to obtain an estimate of the impact of inbreeding under each environmental class. The ID estimates are therefore to be considered as nested within the environmental classes. The calculation was repeated for each stored iteration from the Gibbs sampler implemented as described in this paragraph in order to obtain the posterior distribution of predicted values. Post–burn-in posterior means were used as point estimates, and posterior standard deviations as the corresponding measure of uncertainty. This procedure was carried out using a custom R code, and applied to all five classes of environmental indicator, the three measures of inbreeding, and the four traits, using sampled solutions from the model implemented in the second step.

#### Inbreeding depression and comparison between extremes

To simultaneously assess the effects of environment and inbreeding, we calculated the average genomic inbreeding coefficient, using F_ROH_, for all cows, as well as for the 5% least inbred, and the 5% most inbred individuals. Predicted productive performances were obtained in the same manner as before, across the five environmental classes but specifically for cows within each of these three inbreeding groups. Line plots were generated to illustrate the combined influence of environmental conditions and extreme levels of inbreeding. All graphs were produced using R-package ‘ggplot2’ [44].

## Results

Descriptive statistics for the analysed traits are reported in Table 1. Daily yields average 37.9 kg of milk, with 1.48 kg of fat and 1.29 kg of protein. SCS averaged 2.25.

**Fig. 1:**
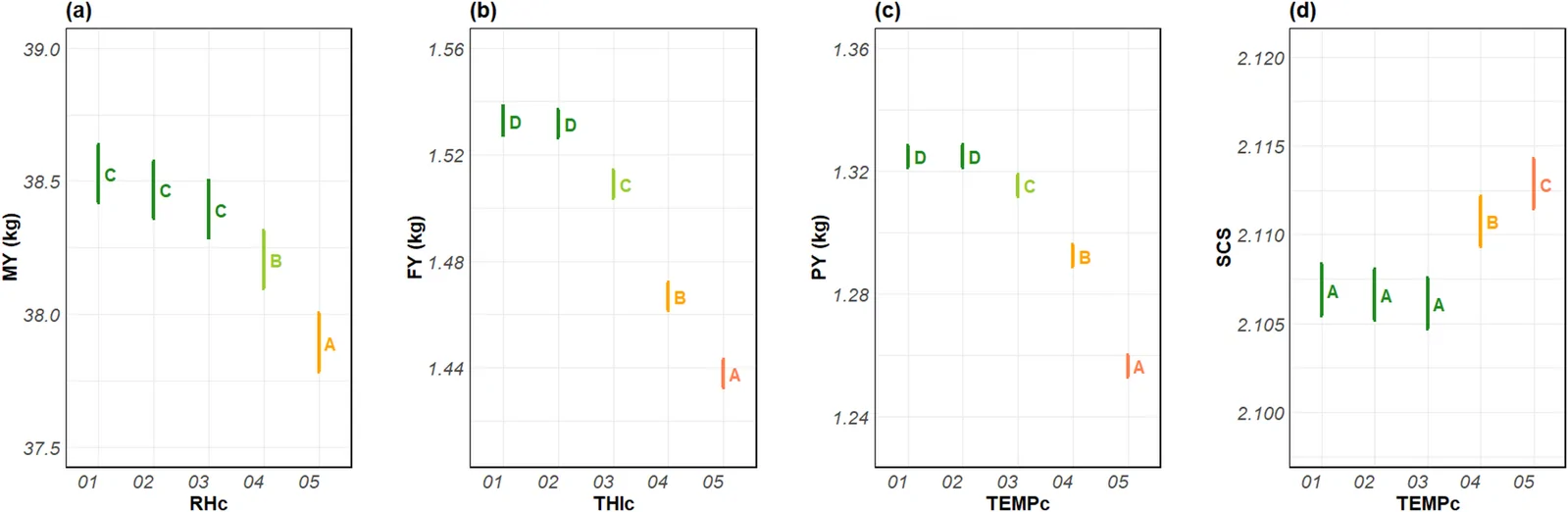
Environmental effects on milk, fat, protein and somatic cell score across environmental classes. Least Square Means productive values estimated according to the respective classes of the environment with the strongest effect for each trait: **a** milk yield (MY) by relative humidity (RHc); **b** fat yield (FY) by temperature humidity index (THIc); **c** protein yield (PY) by temperature (TEMPc); and **d** somatic cell score (SCS) by temperature (TEMPc). These plots illustrate how animals performed across the environmental gradient

The descriptive statistics for the environmental descriptors across the five classes considered are reported in Table 2. The quintile values used to allocate the records in classes based on the environmental descriptors are reported in Additional file 1 Table S1. Weekly TEMP ranged from − 1.5° to 34.2°C, RH ranged from 25.3 to 100%, while THI ranged from ~ 30 to ~ 85 points. It is worth noting that a value of THI above 72 was recorded for more than 20% of the test-days (with the 80th percentile being 73.3). Descriptive statistics for the inbreeding measures are reported in Table 3. The mean number of equivalent complete generations was 8.72 (SD = 1.34), indicating relatively deep and homogeneous pedigree information among phenotyped animals. The descriptive statistics for the traits analysed and the inbreeding measures are reported in Additional file 1 Tables S2 and S3, respectively. The F-statistic values from the ANOVA are presented in Table 4. Results showed that all three environmental variables had significant effects on the traits studied. Except for the effect of RH on SCS, all other environmental variables were highly significant (*P* < 0.01). The table highlights the environmental variable with the strongest effect on each trait. Based on these findings, the most influential environmental variable for each trait was selected to estimate inbreeding effects under different environmental conditions. These were RHc for MY, THIc for FY and TEMPc for PY and SCS, respectively. Least squares mean estimates obtained for each of the four traits, considering the most significant environmental indicator, are presented in Fig. 1.

**Table 2:** Descriptive statistics and threshold values for each class of temperature (TEMP, °C), relative humidity (RH, %), and temperature–humidity index (THI, score)

	Class	N	Mean	SD	Minimum (lower bound)	Maximum (upper bound)
TEMP, °C	1	81,767	8.24	1.86	− 1.50	10.80
	2	83,796	13.31	1.29	10.90	15.50
	3	81,998	17.64	1.25	15.60	19.80
	4	82,465	22.40	1.58	19.90	25.00
	5	84,448	26.97	1.27	25.10	34.20
RH, %	1	82,500	54.24	5.19	25.30	60.60
	2	83,017	64.97	2.36	60.70	68.80
	3	82,841	72.49	2.07	68.90	76.00
	4	83,008	79.96	2.35	76.10	84.20
	5	83,148	90.01	3.86	84.30	100.00
THI, score	1	82,786	47.82	3.32	29.97	52.36
	2	83,017	56.27	1.99	52.36	59.63
	3	82,880	62.86	1.91	59.63	66.24
	4	82,875	69.95	2.13	66.24	73.35
	5	82,956	75.80	1.72	73.35	85.44

Minimum and maximum values indicate the observed range of each environmental variable. The 20%, 40%, 60%, and 80% percentiles (quintiles) define class boundaries

**Table 3:** Descriptive statistics for the inbreeding measures

Measure	Mean	SD	5th percentile	95th percentile
F_PED_	7.48	2.00	4.90	10.90
F_GRM_	27.46	2.89	23.35	32.31
F_ROH_	0.17	0.03	0.12	0.22

Summary statistics are shown for three measures of inbreeding: pedigree-based coefficient (F_PED_, %), genomic relationship matrix diagonal (F_GRM_, %), and runs of homozygosity (F_ROH_, proportion). Mean, SD, 5th, and 95th percentiles are provided

**Table 4:** ANOVA F statistics for climatic environmental effects on production traits

trait	Environmental indicator		
	TEMPc	RHc	THIc
Milk yield	29.95	31.73*	31.53
Fat yield	334.34	66.54	335.66*
Protein yield	369.45*	72.56	365.57
Somatic cell score	29.98*	3.20	29.01

F-statistics from ANOVA of climatic environmental effects on milk yield, fat yield, protein yield, and somatic cell score (SCS) are shown. Environmental indicators include temperature (TEMPc), relative humidity (RHc), and temperature-humidity index (THIc). Flag (*) for the F-value indicate the environmental variable with the strongest effect on each trait, used to evaluate inbreeding-by-environment interactions

### Inbreeding effects

**Fig. 2:**
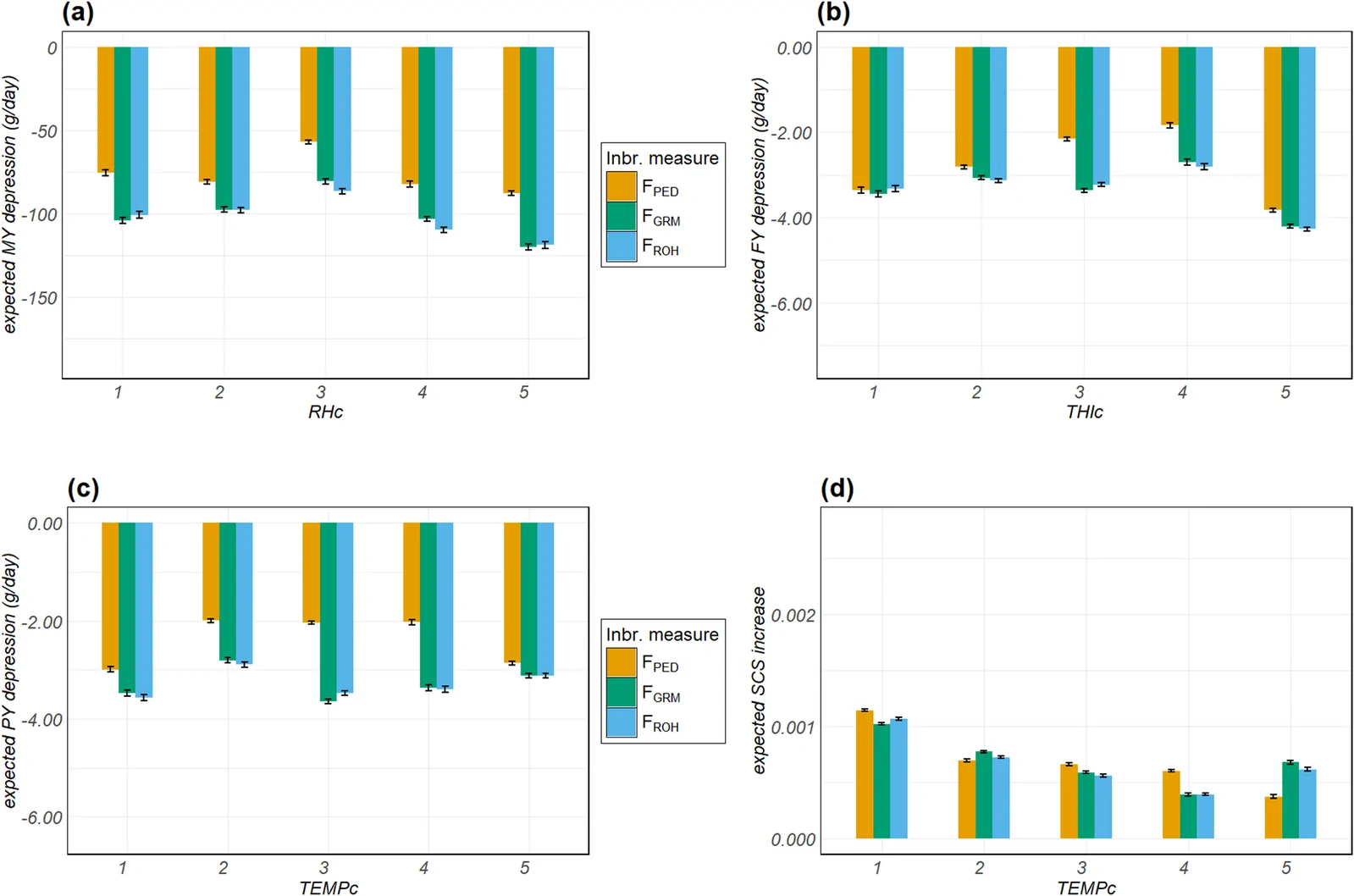
Inbreeding effects across environmental stress classes for four dairy productive traits. Estimated effects of inbreeding, measured with F_PED_, F_GRM_ or F_ROH_, and 95% confidence intervals, across varying classes of environments: **a** milk yield (MY) by relative humidity (RHc); **b** fat yield (FY) by temperature humidity index (THIc); **c** protein yield (PY) by temperature (TEMPc); and **d** somatic cell score (SCS) by temperature (TEMPc). Estimates represent expected changes in each trait for a 1% increase in inbreeding, scaled to match the pedigree-based measure for comparability. Error bars correspond to 95% confidence intervals

Slopes of the inbreeding effects across environment classes measured using F_PED_, F_GRM_, or F_ROH_ are shown in Fig. 2a–d representing MY, FY, PY, and SCS, respectively. The slope values indicate the expected change in each trait for a 1% increase in inbreeding, with measures rescaled to match the mean and standard deviation of F_PED_. The plots for the production traits were scaled so that the y-axis range corresponds to 0.5% of the average value of each trait, ensuring that the vertical bars more clearly reflect the magnitude of inbreeding effects. SCS, on the other hand, was plotted on a rescaled axis because the inbreeding effects were very small relative to the trait mean, which improved readability. Overall, all estimated inbreeding effects were statistically different from zero. For the production traits (MY, FY, and PY), ID was sizable across all types of inbreeding measures and environmental classes, with negative slopes indicating a decrease in trait performance; these slopes corresponded to approximately 0.25% of the trait’s average for each 1% increase in inbreeding. For SCS, inbreeding effects were statistically significant but small, with the estimated increase associated with inbreeding being less than 0.05% of the trait’s mean.

When comparing inbreeding measures, genomic inbreeding (F_GRM_ and F_ROH_) generally captured larger depression effects across most traits and environmental classes, including MY and PY under all environmental classes and FY in more stressful conditions (classes 4 and 5). For SCS, no clear pattern emerged when comparing genomic measures to F_PED_, and the effects were similar regardless of the measure. Additionally, little difference was observed between F_GRM_ and F_ROH_, indicating that both genomic inbreeding measures provided comparable estimates of ID in this study. Finally, F_PED_ appeared to show smaller effects but with larger differences between the EL classes.

### Loss in production due to inbreeding conditional on the environmental load

**Fig. 3:**
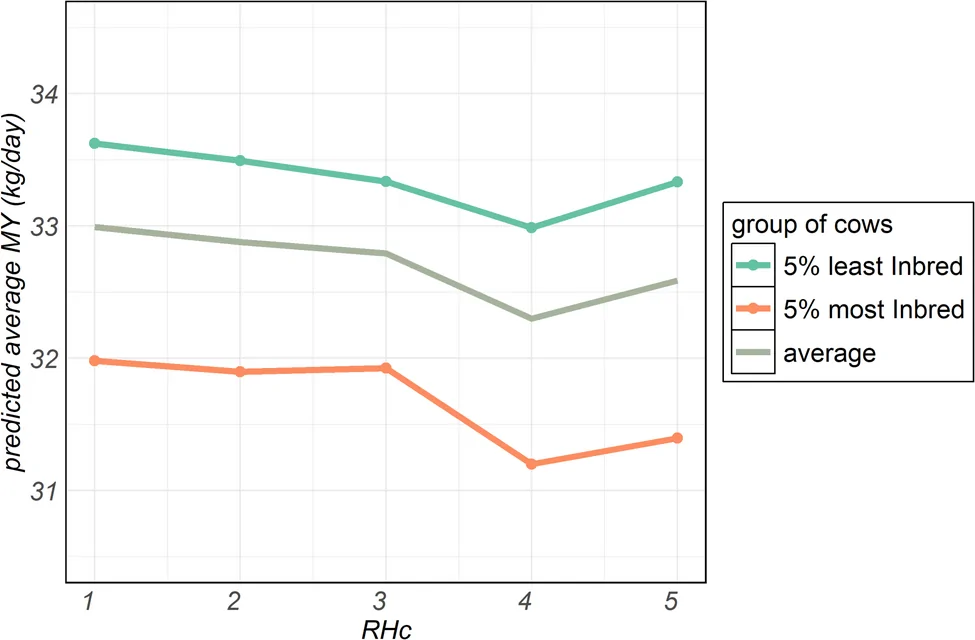
Predicted milk yield (MY) across relative humidity classes (RHc) for cows with three different levels of inbreeding. Predicted MY for cows grouped into three levels of genomic inbreeding five RHc. The figure illustrates that higher inbreeding is associated with clear losses in milk production, and these losses intensify as environmental stress (high RH) increases. The divergence between low- and high-inbreeding cows widens in the upper humidity classes, highlighting a biological interaction between inbreeding and the ability to maintain production under heat-associated stress

**Fig. 4:**
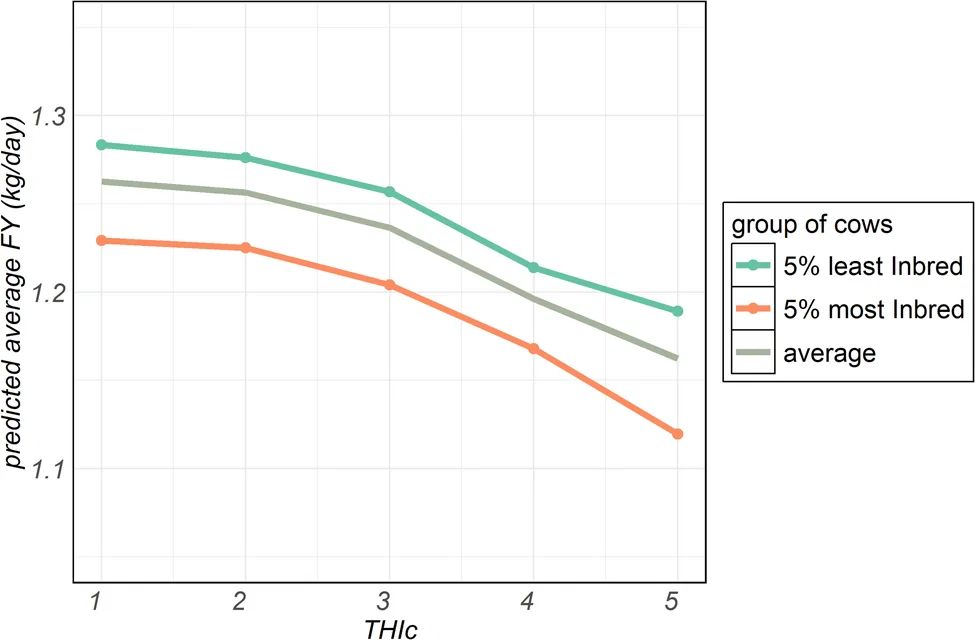
Predicted fat yield (FY) across temperature humidity index classes (THIc) for cows with three different levels of inbreeding. Predicted FY for cows at three levels of genomic inbreeding across five THIc. As THI increases FY declines sharply, with the steepest reductions observed in highly inbred cows. Differences between inbreeding groups become more evident in the highest THI classes, demonstrating that inbreeding amplify heat-related drops in fat production. This pattern indicates that inbreeding depression intensifies under heat stress

**Fig. 5:**
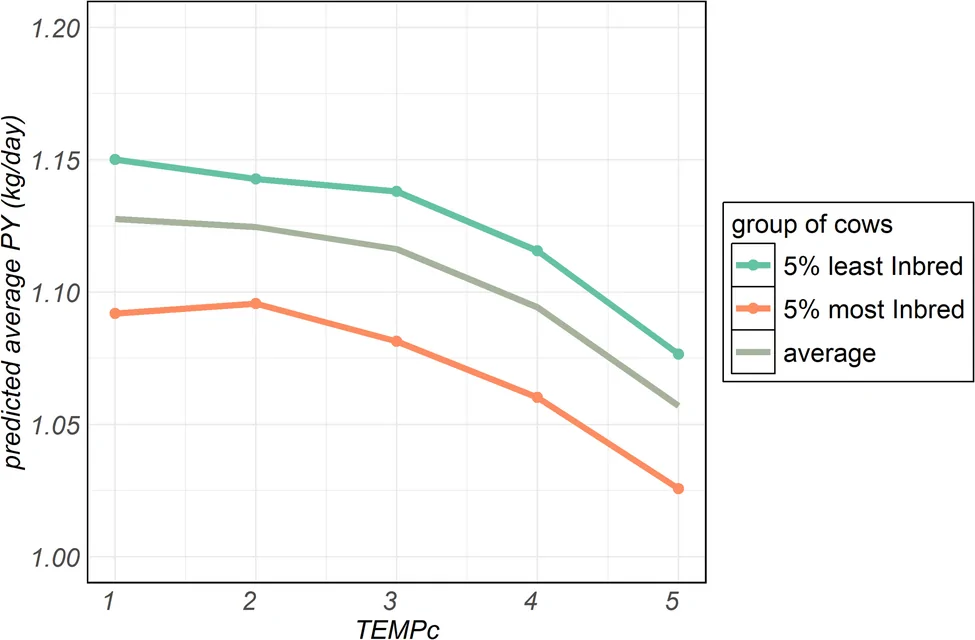
Predicted protein yield (PY) across temperature classes (TEMPc) for cows with three different levels of inbreeding. Predicted PY for cows with low, medium, and high inbreeding across increasing TEMPc. PY decreases as temperature rises, with moderately larger declines in more inbred cows. However, compared to milk and fat yield, the interaction between inbreeding and environmental stress appears less pronounced, consistent with the study’s results. These findings suggest that although PY is negatively affected by both temperature and inbreeding, the degree to which environmental stress amplifies inbreeding depression may be smaller for this trait

**Fig. 6:**
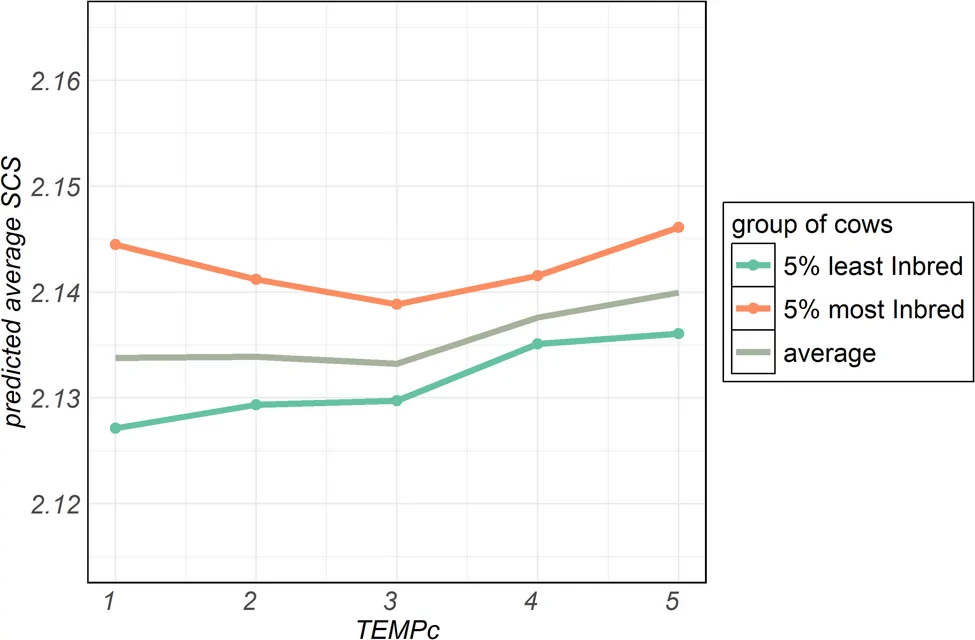
Predicted somatic cell score (SCS) across temperature classes (TEMPc) for cows with three different levels of inbreeding. Predicted SCS for cows with three levels of genomic inbreeding across TEMPc. SCS increases with rising temperatures, reflecting greater under-health challenges under heat stress. Higher inbreeding is associated with slightly greater SCS, although the amplification of inbreeding effects across temperature classes is limited compared to production traits

The realized production losses are shown in Figs. 3, 4, 5, and 6 for MY, FY, PY, and SCS, respectively. For SCS, an increase in the score is interpreted as a loss. Each figure presents the trait change (y-axis) across EL (x-axis) for cows with F_ROH_ at the mean (grey line), the 5th percentile (green line), and the 95th percentile (orange/red line). The 5th-percentile and 95th-percentile trajectories represent the most outbred and most inbred animals, respectively. Across all traits, higher EL was generally associated with poorer performance, in line with the model results shown in Fig. 1. Losses were strongest and most consistent for fat and PY, which displayed a nonlinear decline with increasing heat stress. MY showed a partial rebound at the highest environmental-load class (RHc 5 versus 4). SCS increased with EL, indicating deteriorating milk quality under heat stress. In every plot, the lines for the outbred and inbred groups are not perfectly parallel to the average inbreeding line, as a result of the inbreeding effects being different across classes of EL, as reported in Fig. 2. Despite the rebound, the groups show a stronger spread for MY under RHc classes 4 and 5, followed by classes 1 and 2, with class 3 showing the narrower differences. This is consistent with the F_ROH_-driven depression values shown in Fig. 2a, where the depression is sensibly stronger under RHc classes 4 and 5. Likewise, the plot for FY shows the largest spread under THIc 5, consistent with the larger depression shown in Fig. 2b. A similar, albeit milder pattern is reported for PY (Fig. 5), which is also consistent with the environmental determination of ID for F_ROH_ (Fig. 2c).

ID generally increased with greater EL, with the strongest evidence observed for MY and FY. The most stressful conditions corresponded to environmental-load classes 4 and 5, although some trait-specific differences were apparent.

## Discussion

In this study, we evaluated the impact of ID on MY, FY, PY, and SCS in the Italian Holstein population, under varying levels of environmental stress. Inbreeding was estimated using pedigree information (F_PED_) as well as genomic data (F_GRM_, F_ROH_). Environmental stress was quantified from 7-day averages of TEMP, RH, and temperature-humidity index. In general, the sample of 24,606 cows used can be considered representative of the Italian Holstein population. Yields were similar to the general population (for a full report, see http://bollettino.aia.it) and inbreeding values were comparable to what reported by Ablondi et al. [16] and Panetto et al. [31] on subsets of the same population, and also by Lozada-Soto et al. [45] in the Holstein population of the US.

### Framework

Heat tolerance is typically evaluated using reaction norms models, however in this study we did not use that approach, in favor of a less explicit but more interpretable one. First, we assessed the degree of stress due to heat as manifested in production loss. The least squares means reported in Fig. 1 aim at assessing this impact. Second, in light of the impact assessed, we estimated ID for the production traits conditionally on the climatic classes. ID estimates conditional on heat stress level illustrates how EL modulates the expression of ID. The within-class estimates make this modulating effect explicit, showing that the impact of inbreeding on production traits is not uniform but depends on the environmental conditions under which animals perform. While simplistic, this approach warrants ease of interpretation, in addition to a straightforward application of the results. Indeed, the loss in productivity can be easily assessed as due to heat, inbreeding and both: breeders and producers are provided with a schematic representation of the losses and therefore of the parameters (climate or inbreeding) to control.

### Environmental effects and environmental load

Climatic variables taken from weather stations and used in the ANAFIBJ routine evaluation for the heat tolerance index (IHT; [11]) were used as environmental descriptors in this study. These variables show a representative picture of the temperate Mediterranean climate that cows undergo in the country.

For each trait, we selected the environmental indicator that showed the largest impact on average performance, and this was used to categorize subsequent analyses of inbreeding effects. We aimed to identify climatic conditions that classify the environment as either benign or stressful [46], using the resulting EL as the metric. Although direct measures of fitness were not available, production traits were used as proxies to characterize the environment and quantify the EL experienced by the cows. Thus, we compared the model fit from the ANOVA using alternative climatic variables for measuring the EL. Table 4 highlights the environmental variable with the strongest effect on each trait. Our estimates of average productivity losses under environmental stress fall mostly within ranges reported previously by other authors. Bernabucci et al. [7] reported daily MY losses of 0.91–1.27 kg in the Italian Holstein population, values somewhat larger than those observed here. Their FY and PY losses (0.02–0.07 kg and 0.02–0.10 kg, respectively) overlap with our results. Santana et al. [47] similarly reported substantial MY declines in Brazilian Holsteins, consistent with our findings, although they observed a reduction in SCS in more stressful environments. In a North American Holstein dataset, McWhorter et al. [12] estimated decreases of approximately 0.9 kg, 0.3 kg, and 0.3 kg in daily milk, fat, and protein yields, respectively, for animals in the most stressful environments.

MY was evaluated using RHc as the environmental-load indicator, while FY used THIc. MY showed minor discrepancies between the two models as to whether class 4 or 5 was the harshest. These discrepancies likely arise from differences in the environment indicators used and from how they were incorporated into the models, which may affect their sensitivity to the signal in the data. A primary contributor may be reliance on weather-station data rather than on-site indoor measurements; differences in housing structure and cooling systems can decouple external weather from the actual barn microclimate. The pattern observed for MY is consistent with the findings of Lovarelli et al. [48], who reported similar responses of MY and cow behavioral traits under high-THI conditions in a related population. In contrast, this trend is not evident for FY across THI classes. Under increasing thermal load (THIc 3–5, corresponding to moderate-to-severe heat stress), FY shows a more systematic response, with a non-linear pattern that becomes markedly detrimental under the highest stress levels (THIc 4–5). In this scenario, ID is detectable across all environments but becomes statistically relevant only under THIc 5, regardless of the inbreeding measure considered (F_PED_, F_GRM_ or F_ROH_). The observed pattern, worse environmental conditions amplifying ID, aligns with expectations for genotype-by-environment interactions.

### The environmental load modulates the expression of inbreeding depression

Comparable livestock studies are scarce, so findings are more readily compared with work on wild populations. Armbruster and Reed [46] reviewed 34 studies across taxa and reported the same pattern in most cases, typically using direct fitness measures. Although our focus is on production and health traits rather than classical fitness metrics, the relationship is plausible: increased inbreeding raises the probability of expressing recessive deleterious alleles, which are more likely to be revealed under heat-stress conditions and manifested as losses in production (MY, FY, PY) and health (SCS).

Evidence of ID in livestock has also been extensively documented independently of environmental modulation. Genomic inbreeding has been associated with reductions in MY, FY and PY [16] decreased stayability between lactations [31], and impaired fertility expressed as increased probability of re-insemination after first service [15]. These results confirm that the biological mechanisms underlying ID are already measurable under average management conditions and may become amplified when animals are exposed to environmental challenges.

The use of climatic variables to quantify EL, along with observed irregularities across classes, highlights a key challenge noted by previous authors. Accurate measurement of stress and EL is critical for reliable inference of genotype-by-environment interactions. Additionally, part of the difficulty in interpreting inbreeding effects may arise from the way inbreeding itself is quantified. F_PED_ depends strongly on pedigree depth and completeness, and differences in genealogical knowledge across populations can influence both the magnitude and the apparent pattern of ID [31]. Our approach relies on two assumptions: first, that stressful conditions are those that reduce fitness [28, 49]; second, that production and health traits in livestock serve as proxies for fitness akin to that measured in wild populations. With the definition of EL becoming increasingly complex, fitness should not be the only metric for assessing stress outcomes. Any measure of outcomes such as production, health, or fitness may reflect a combination of multiple factors, not all of which contribute uniformly or in the same direction. Thus, the quantification of stress outcomes is becoming increasingly complex. This was already hypothesized by Armbruster and Reed [46], which attributed the lack of ID to factors that can affect one component of the lifecycle while leading to a change in the others, of opposite sign. As an example, Vega-Trejo et al. [50] showed that dietary restriction was not a factor that would permanently create the conditions for stronger ID, although being a clear factor of stress. In their study in mosquitofish (*Gambusia holbrooki*), they attributed this lack of regulation to some compensatory growth. In our study, lower production under heat stress might be an indication of stress, but other factors could contribute to the opposite direction. These factors include diet adjustments and cooling systems, with the latter potentially explaining the observed rebound in MY between relative-humidity classes 4 and 5. This highlights the need for further research to accurately define stressful conditions using traits and environmental measures that are not compounded by multiple, inconsistently contributing factors.

Actually, the learning of stress from the data itself might collide with assumptions from literature. Pemberton et al. [28] pointed out some discrepancies found in their study on Soay sheep. The generalized lack of fitness might mask ID itself, shrinking the variance due to this effect. For example, female survival was found to be more strongly impacted by ID in high-density conditions, yet the trait itself was not significantly affected by population density alone. Similar patterns have been reported in invertebrate species such as *Arianta arbustorum* (snails) and *Tribolium castaneum* (red flour beetle), where ID was primarily observed under conditions associated with the highest fitness levels [51, 52]. It should be noted that higher fitness conditions (more benign) and higher ID were those with high environmental variability, not necessarily constantly unfavourable conditions.

This raises further considerations regarding the appropriate definition of stress level or EL. First, the intensity of stress experienced may not always be sufficient to clearly demonstrate the expected relationship, greater depression in harsher environments, as suggested by Fox and Reed [49]. In this study, for example, FY showed a greater F_ROH_-associated depression only under the most severe heat stress condition (THIc 5, with THI above 73.3). However, the effects of heat stress on FY have become evident already at THIc 4 and 5 (THI above 66.2).

Consequently, without records of THIc 5, estimating the true impact of heat stress on inbreeding-by-environment interactions would have been more challenging. Additionally, most studies (including ours) define stress or EL as a single variable, while stress should be better defined as a longitudinal, high-dimensional sum of factors contributing to it. While this is well established for the study of additive genetic effects under different environments [53–55], this is also supported by literature on wild populations [46, 49] when dealing with fitness and its components thereof. As evidence accumulates regarding the genetic architecture of heat tolerance, particularly that selection for this trait may compromise genetic progress for production, a potential strategy would be to focus on non-additive effects. This could be achieved through selection based on overall genetic value or via controlled matings, thereby potentially improving resilience without compromising production gains.

When considering additional stress indicators, it is important to focus not only on the average values but also on the variation observed under specific environmental conditions. Results in Armbruster and Reed [46] illustrate how trait variance can be significantly affected by the proportion of individuals unable to survive or reproduce, leading to a reduction in variance that diminishes statistical power to detect inbreeding effects and results in their underestimation. In our study, phenotypic variance appears inconsistent across different EL classes (Table S1). Although this pattern seems to roughly correspond to the magnitude of ID, these observations highlight the need for further targeted analyses to elucidate the relationship between phenotypic variance under certain environments and the ID they produce.

## Conclusions

The present study shows that the magnitude of inbreeding depression could be modulated by the stress generated by environmental conditions, namely climatic conditions. This consideration is important when evaluating the impact of inbreeding on relevant traits, as the absence of stress conditions may conceal the true extent of inbreeding effects. Conversely, the finding that heat tolerance is influenced by inbreeding highlights its relevance for assessing economic and welfare losses resulting from heat stress, which are likely to become increasingly prevalent in the future. Overall, the results of this study indicate that autozygosity contributes to heat sensitivity in lactating dairy cows. In addition to ongoing selective breeding programs aimed at improving heat tolerance, managing levels of homozygosity may represent a complementary strategy to further enhance resilience to environmental load. This could be achieved through selection based on overall genetic value or via controlled matings, thereby potentially improving resilience without compromising production gains.

## Supplementary Information

Below is the link to the electronic supplementary material.

Supplementary Material 1

Supplementary Material 2

## Data Availability

Research data are not shared.
