# Supplementary material for: Heat-load-dependent inbreeding depression for production traits in Italian Holstein cattle
Source: Genet Sel Evol. 2026 Jul 19;58:60. doi: 10.1186/s12711-026-01069-2 (PMC13501847; doi:10.1186/s12711-026-01069-2)
Supplement: Supplementary file 1 [file 12711_2026_1069_MOESM1_ESM.pdf]

Table S1 Threshold values (quintiles) used to categorize temperature (TEMP, °C), relative humidity (RH, %), and temperature–humidity index (THI) into five equally sized classes.

**Threshold**	**Environment**		
	**TEMP**	**RH**	**THI**
Minimum	-1.50	25.30	29.97
20%	10.90	60.70	52.36
40%	15.60	68.90	59.63
60%	19.90	76.10	66.24
80%	25.10	84.30	73.35
Maximum	34.20	100.0	85.44

Minimum and maximum values indicate the observed range of each environmental variable. The 20%, 40%, 60%, and 80% percentiles (quintiles) define class boundaries.

Table S2 Descriptive statistics for milk yield, fat yield, protein yield, and somatic cell score across environmental classes.

	Class	Number of records	Number of cows	Number of HTD classes	Milk yield		Fat yield		Protein yield		Somatic cell score	
					Mean	SD	Mean	SD	Mean	SD	Mean	SD
Temperature	1	81,767	24,395	4,876	38.01	8.91	1.53	0.42	1.32	0.28	2.18	1.85
	2	83,796	24,395	4,907	38.41	9.02	1.53	0.42	1.32	0.28	2.20	1.89
	3	81,998	24,395	4,789	38.18	8.99	1.50	0.42	1.31	0.280	2.21	1.89
	4	82,465	24,395	5,315	37.64	8.89	1.45	0.41	1.28	0.28	2.33	1.92
	5	84,488	24,395	5,124	37.29	8.72	1.41	0.40	1.24	0.27	2.34	1.96
Relative humidity	1	82,500	24,395	5,200	37.40	8.67	1.43	0.40	1.25	0.27	2.27	1.89
	2	83,017	24,395	4,990	37.89	8.87	1.47	0.41	1.28	0.28	2.28	1.92
	3	82,841	24,395	5,058	38.04	8.97	1.49	0.42	1.30	0.28	2.28	1.93
	4	83,008	24,395	4,961	38.18	9.06	1.51	0.43	1.32	0.28	2.22	1.89
	5	83,148	24,395	4,802	37.99	8.99	1.51	0.42	1.31	0.28	2.21	1.89
Temperature-humidity index	1	82,786	24,395	4,922	38.04	8.93	1.53	0.42	1.32	0.28	2.18	1.85
	2	83,017	24,395	4,846	38.42	9.01	1.53	0.42	1.33	0.28	2.19	1.89
	3	82,880	24,395	4,932	38.16	9.00	1.50	0.42	1.30	0.28	2.22	1.88
	4	82,875	24,395	5,350	37.49	8.81	1.44	0.40	1.27	0.27	2.33	1.92
	5	82,956	24,395	4,961	37.41	8.78	1.42	0.40	1.25	0.27	2.34	1.97

Table S3 Descriptive statistics for inbreeding measures across environmental classes.

	Class	F_{PED}				F_{GRM}				F_{ROH}			
		Mean	SD	5th perc.	95th perc.	Mean	SD	5th perc.	95th perc.	Mean	SD	5th perc.	95th perc.
Temperature	1	7.31	1.99	4.70	10.80	27.25	2.87	23.19	32.11	16.30	3.39	11.31	22.13
	2	7.51	2.00	4.90	10.90	27.51	2.88	23.39	32.35	16.62	3.42	11.56	22.48
	3	7.45	2.02	4.90	10.90	27.42	2.89	23.34	32.3	16.51	3.41	11.49	22.38
	4	7.33	2.00	4.70	10.80	27.27	2.89	23.17	32.16	16.31	3.41	11.29	22.21
	5	7.45	1.99	4.90	10.90	27.43	2.85	23.39	32.26	16.52	3.37	11.54	22.34
Relative humidity	1	7.28	2.00	4.60	10.80	27.20	2.86	23 .1	32.07	16.25	3.40	11.23	22.14
	2	7.38	1.97	4.90	10.80	27.36	2.86	23.31	32.2	16.42	3.37	11.46	22.25
	3	7.45	1.99	4.90	10.80	27.44	2.86	23.39	32.23	16.53	3.39	11.53	22.30
	4	7.52	2.01	4.90	11.00	27.51	2.91	23.39	32.37	16.61	3.42	11.56	22.51
	5	7.42	2.02	4.80	10.90	27.37	2.88	23.29	32.27	16.45	3.41	11.43	22.33
Temperature-humidity index	1	7.32	1.99	4.70	10.80	27.26	2.87	23.19	32.12	16.30	3.39	11.31	22.14
	2	7.52	2.00	4.90	11.00	27.51	2.88	23.39	32.36	16.63	3.42	11.55	22.50
	3	7.42	2.02	4.80	10.90	27.39	2.90	23.31	32.27	16.46	3.42	11.43	22.35
	4	7.30	2.00	4.70	10.80	27.24	2.87	23.16	32.13	16.28	3.40	11.29	22.17
	5	7.49	1.98	5.00	10.90	27.48	2.85	23.43	32.31	16.58	3.37	11.61	22.40

Summary statistics are shown for three measures of inbreeding: pedigree-based coefficient (F_{PED}, %), genomic relationship matrix diagonal (F_{GRM}, %), and runs of homozygosity (F_{ROH}, proportion multiplied by 100 to magnify differences). Mean, SD, 5th, and 95th percentiles are provided.
