# Supplementary material for: Heat-load-dependent inbreeding depression for production traits in Italian Holstein cattle
Source: Genet Sel Evol. 2026 Jul 19;58:60. doi: 10.1186/s12711-026-01069-2 (PMC13501847; doi:10.1186/s12711-026-01069-2)

Figure S1 Predicted milk yield across five classes of relative humidity by genomic inbreeding level.

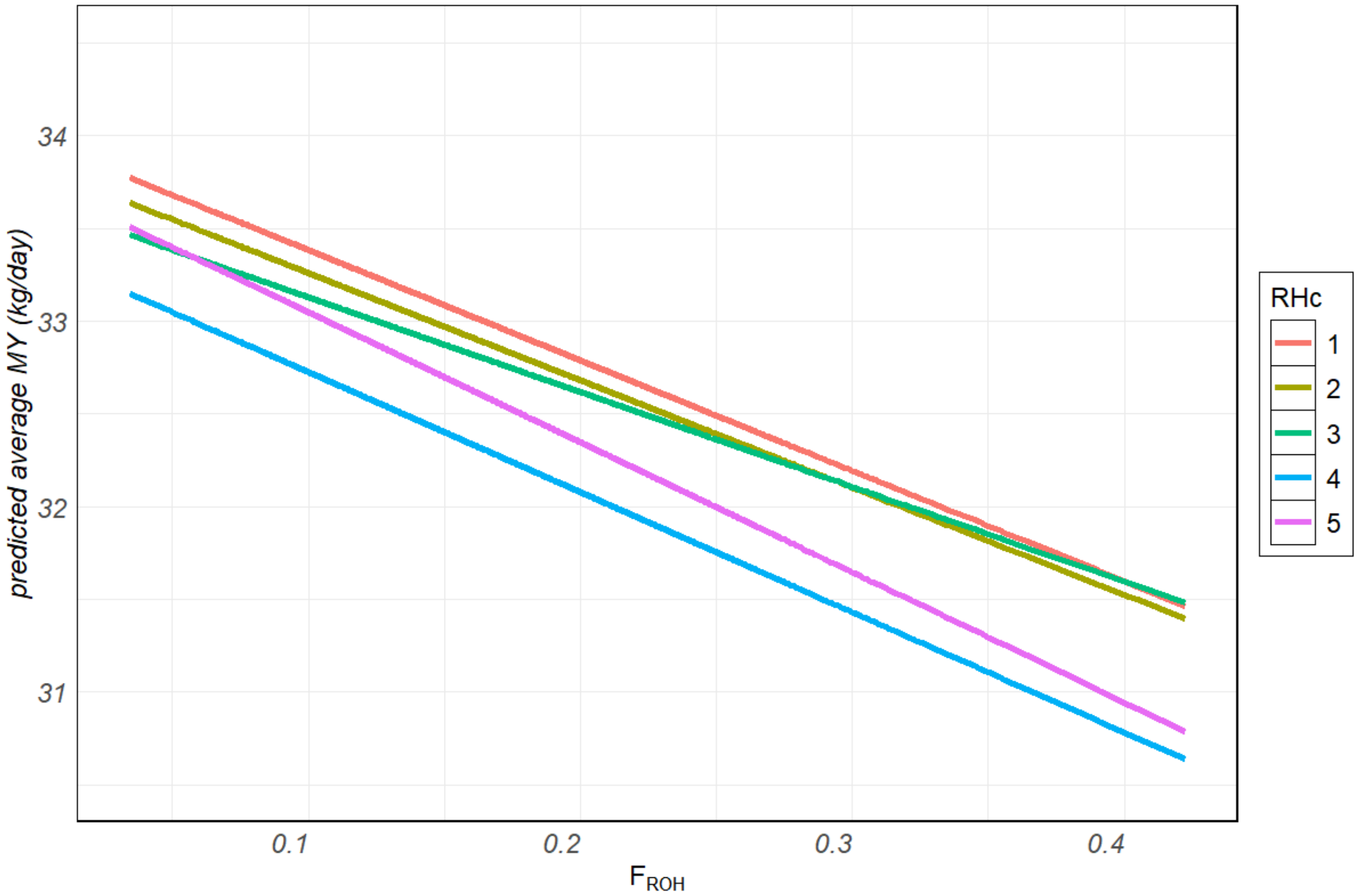

Figure S2 Predicted fat yield across five classes of temperature–humidity index by genomic inbreeding level.

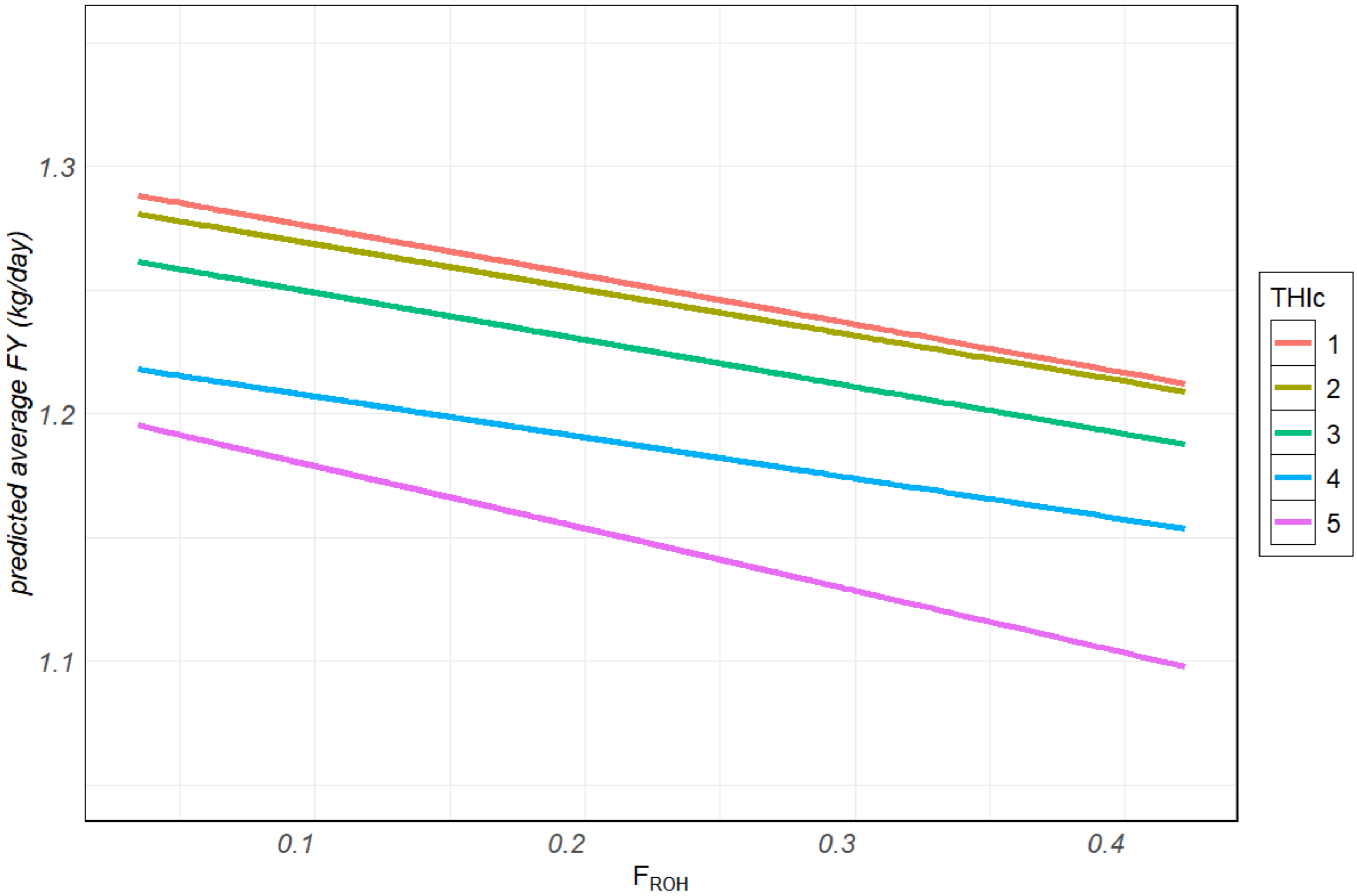

Figure S3. Predicted protein yield across five classes of temperature by genomic inbreeding level.

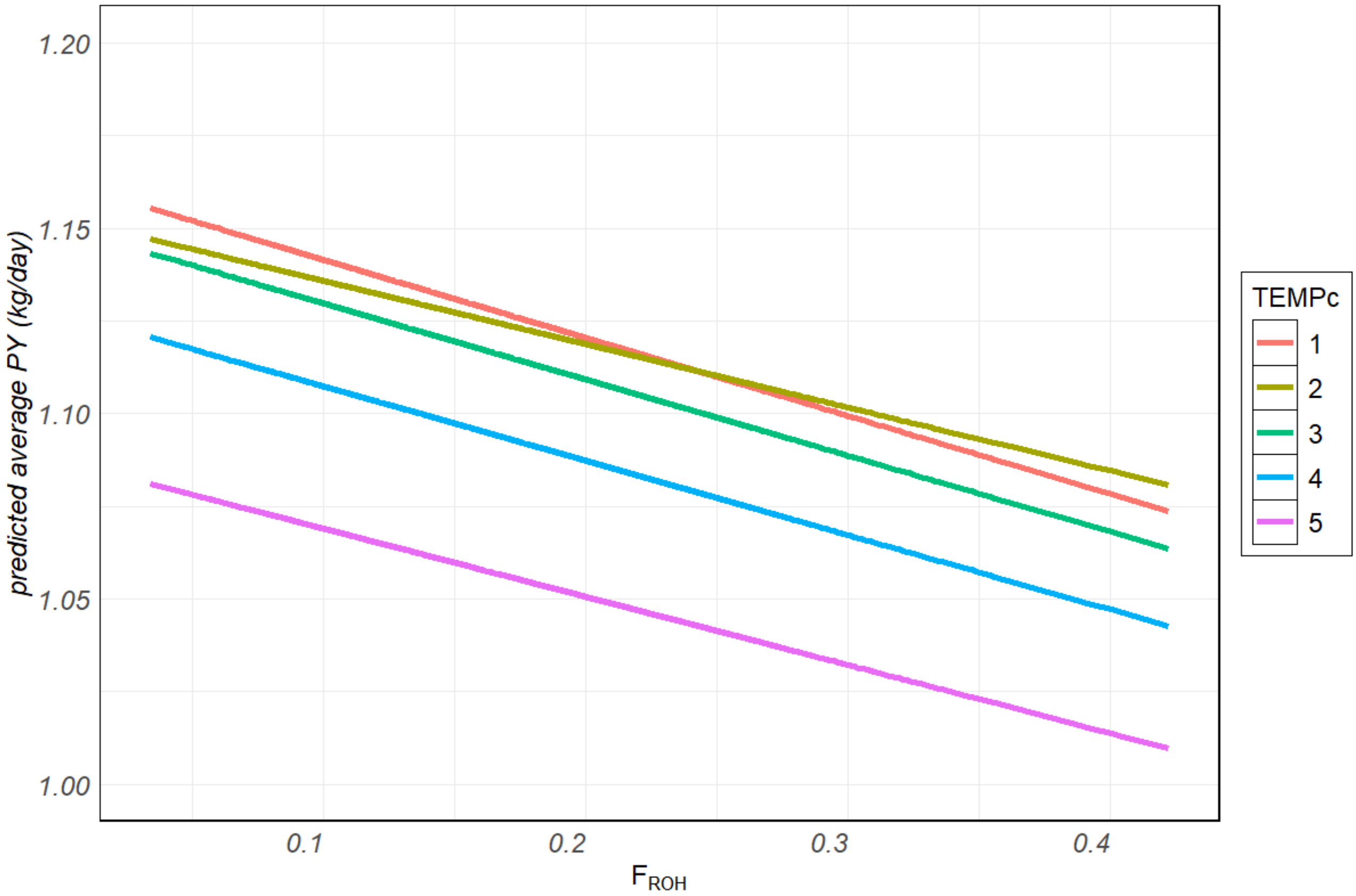

Figure S4. Predicted somatic cell score across five classes of temperature by genomic inbreeding level.

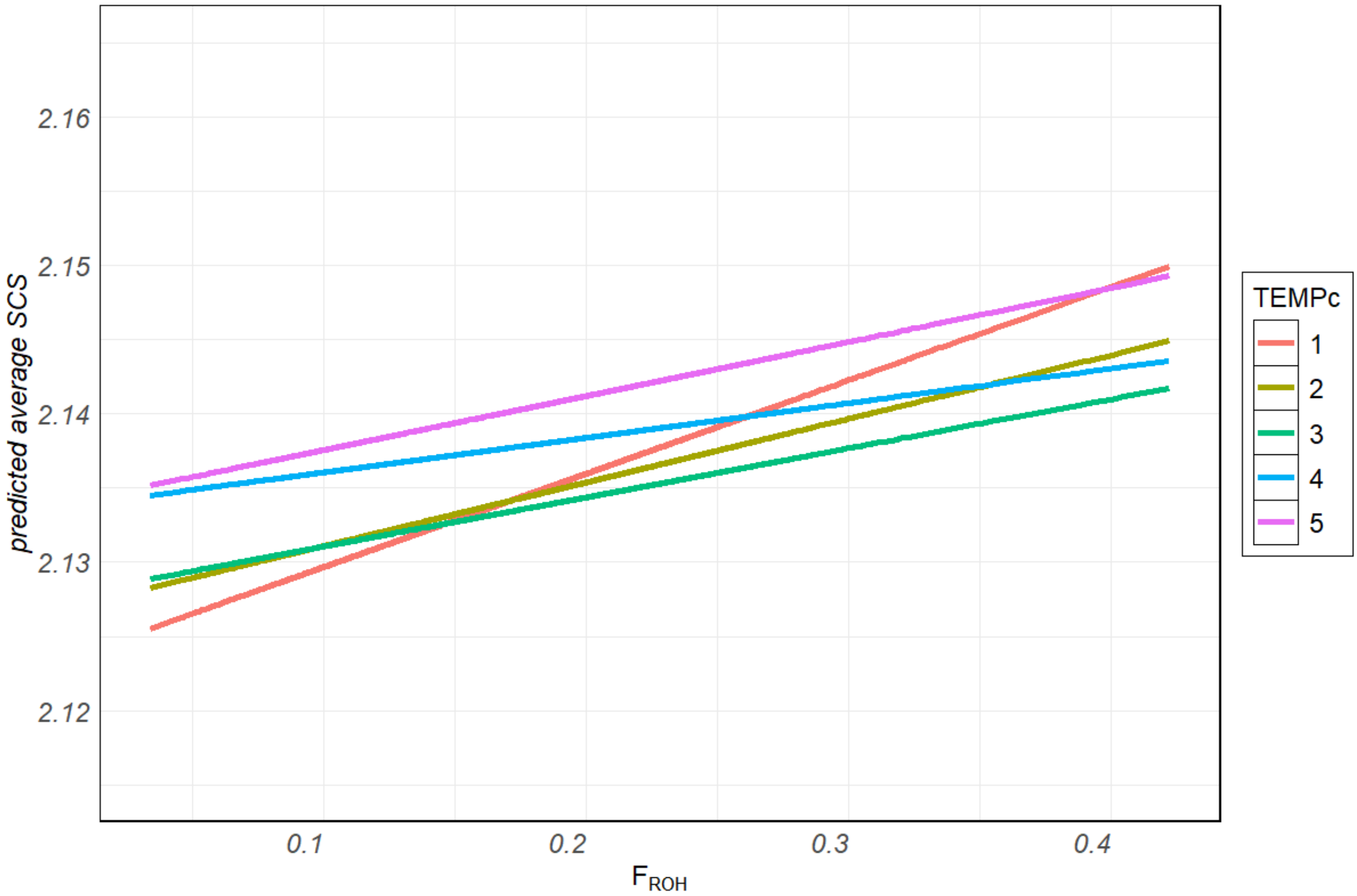

Figure S5 Distribution of milk yield records.

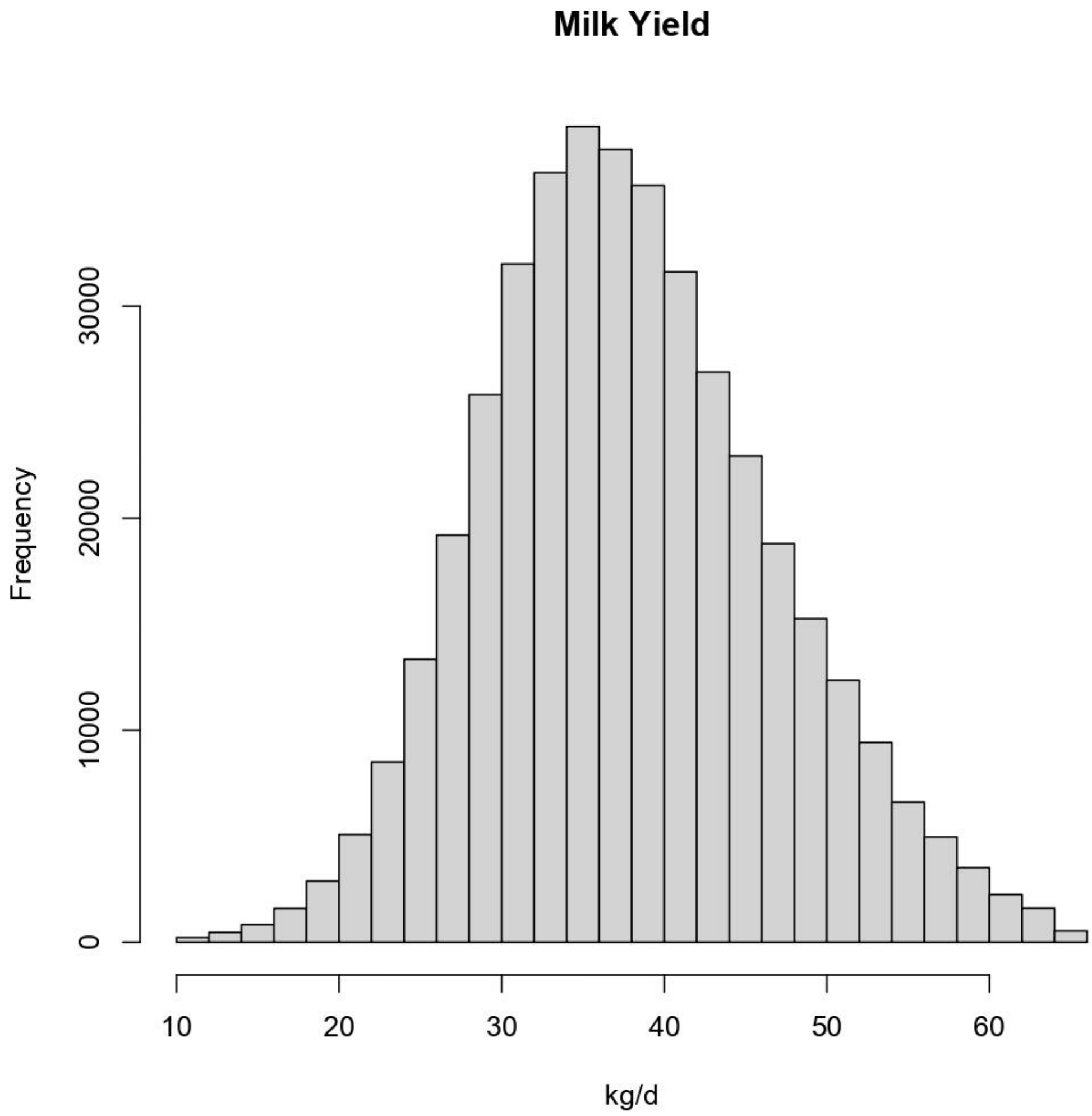

Figure S6 Distribution of fat yield records.

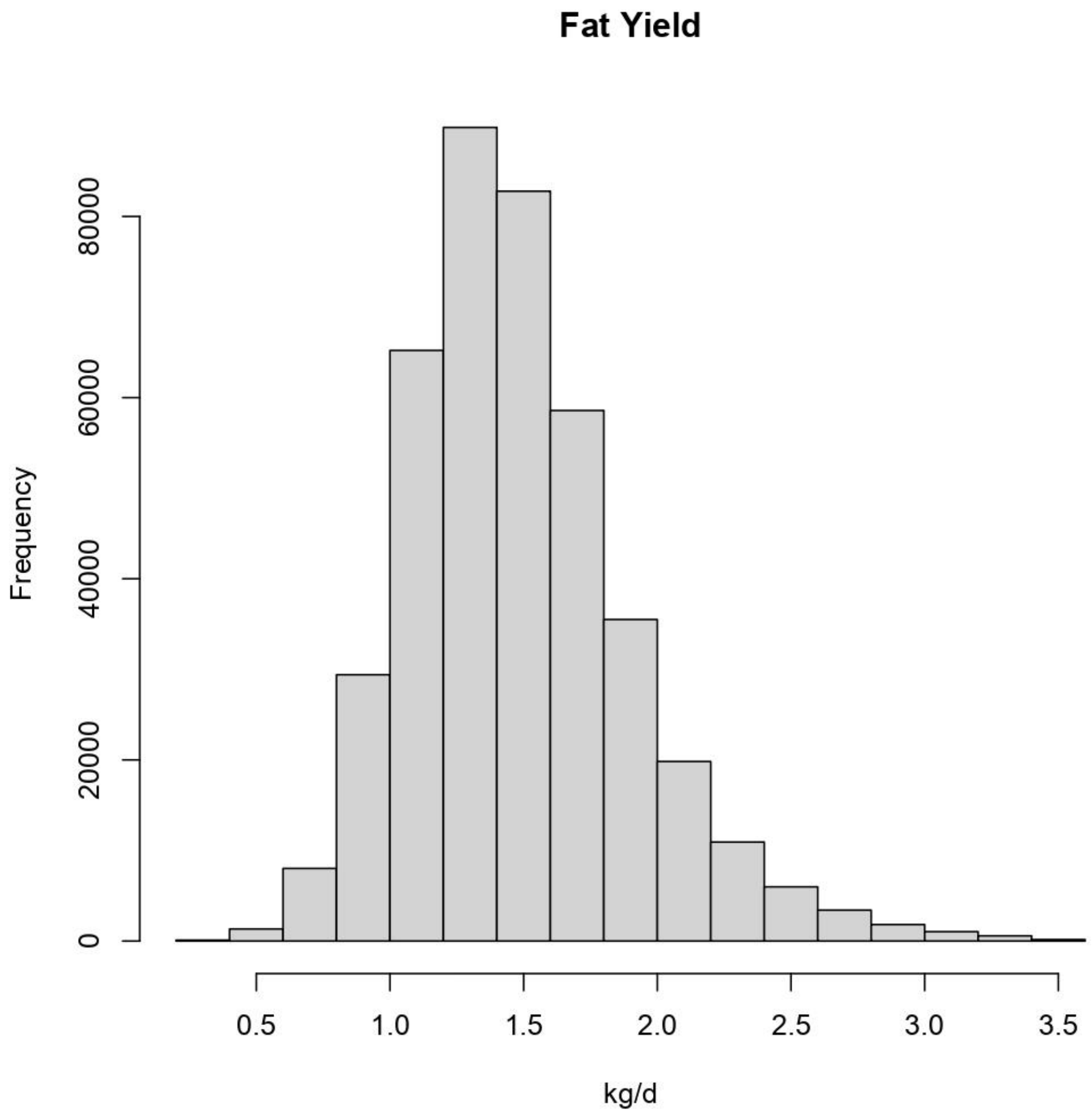

Figure S7 Distribution of protein yield records.

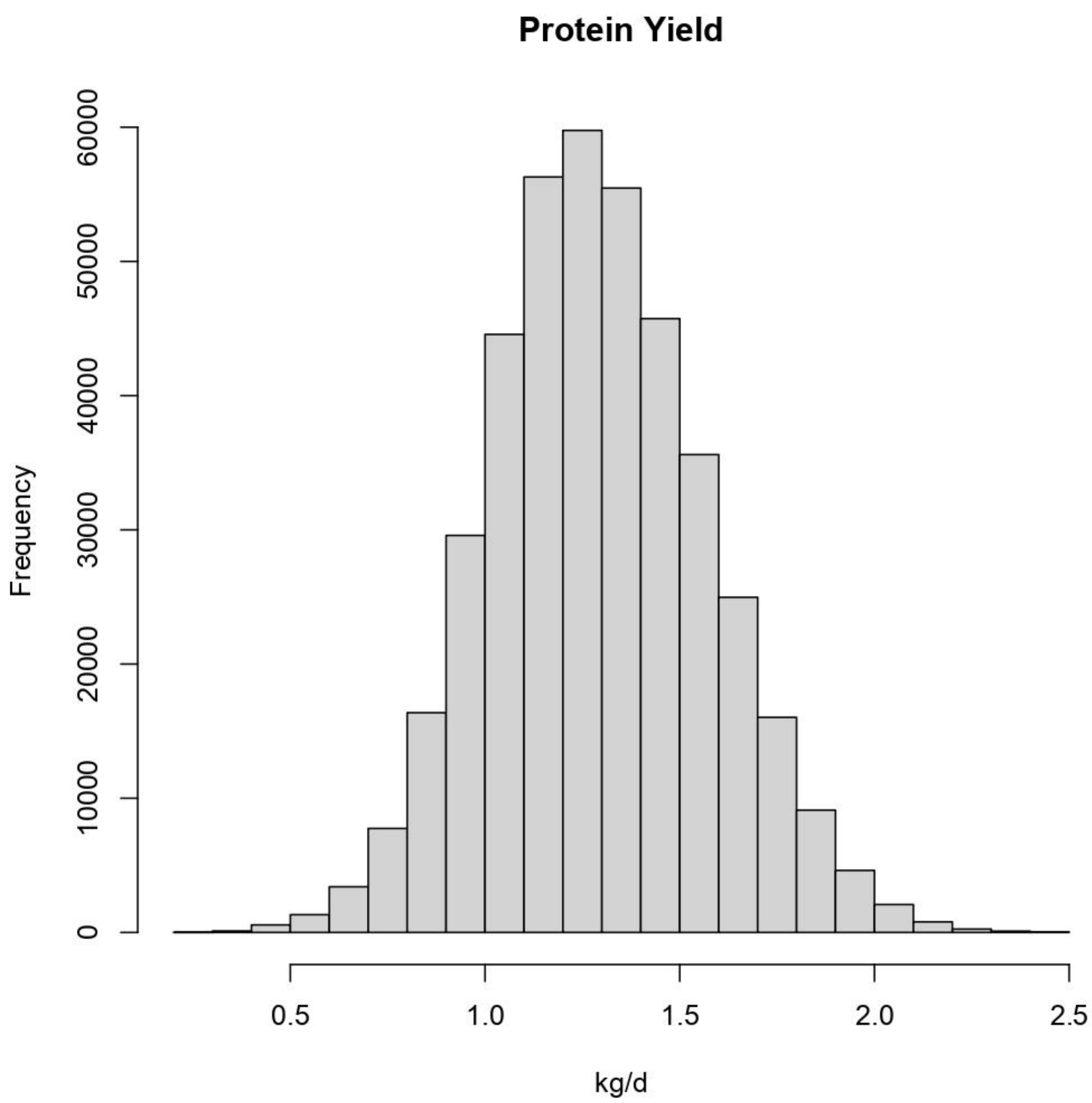

Figure S8 Distribution of somatic cell score records.

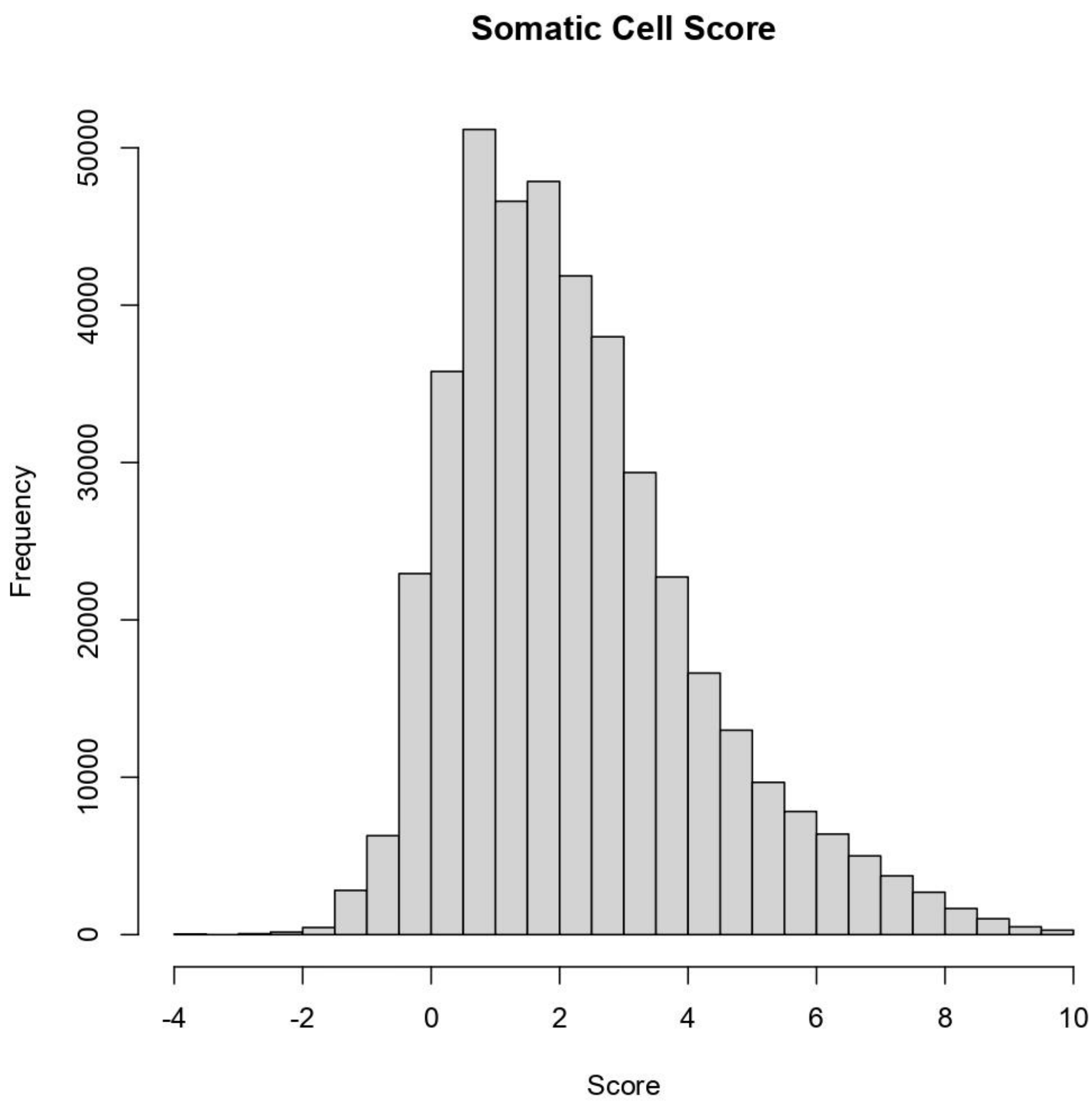

Supplement: Supplementary file 2 [file 12711_2026_1069_MOESM2_ESM.pdf]
